# Advancements in Nucleic Acid Based Therapeutics against Respiratory Viral Infections

**DOI:** 10.3390/jcm8010006

**Published:** 2018-12-20

**Authors:** Kumari Asha, Prashant Kumar, Melvin Sanicas, Clement A. Meseko, Madhu Khanna, Binod Kumar

**Affiliations:** 1 Department of Microbiology and Immunology, Chicago Medical School, Rosalind Franklin University of Medicine and Science, North Chicago, IL 60064, USA; 2 Amity Institute of Virology and Immunology, Amity University, Noida 201303, India; pkumar18@amity.edu; 3 Sanofi Pasteur, Asia and JPAC Region, Singapore 257856, Singapore; melvin.sanicas@gmail.com; 4 Regional Centre for Animal Influenza, National Veterinary Research Institute, Vom 930010, Nigeria; cameseko@yahoo.com; 5 Department of Respiratory Virology, Vallabhbhai Patel Chest Institute, University of Delhi, Delhi 110007, India; madhukhanna@hotmail.com

**Keywords:** nucleic acid therapy, siRNA, ribozyme, aptamers, DNAzyme, influenza virus, RSV, SARS-CoV, adenovirus, antiviral drugs

## Abstract

Several viruses cause pulmonary infections due to their shared tropism with cells of the respiratory tract. These respiratory problems due to viral infection become a public health concern due to rapid transmission through air/aerosols or via direct-indirect contact with infected persons. In addition, the cross-species transmission causes alterations to viral genetic makeup thereby increasing the risk of emergence of pathogens with new and more potent infectivity. With the introduction of effective nucleic acid-based technologies, post translational gene silencing (PTGS) is being increasingly used to silence viral gene targets and has shown promising approach towards management of many viral infections. Since several host factors are also utilized by these viruses during various stages of infection, silencing these host factors can also serve as promising therapeutic tool. Several nucleic acid-based technologies such as short interfering RNAs (siRNA), antisense oligonucleotides, aptamers, deoxyribozymes (DNAzymes), and ribozymes have been studied and used against management of respiratory viruses. These therapeutic nucleic acids can be efficiently delivered through the airways. Studies have also shown efficacy of gene therapy in clinical trials against respiratory syncytial virus (RSV) as well as models of respiratory diseases including severe acute respiratory syndrome (SARS), measles and influenza. In this review, we have summarized some of the recent advancements made in the area of nucleic acid based therapeutics and highlighted the emerging roles of nucleic acids in the management of some of the severe respiratory viral infections. We have also focused on the methods of their delivery and associated challenges.

## 1. Introduction

Many viruses show tropism for cells of the respiratory tract that supports viral entry and subsequent infection. Infection with such respiratory viruses often leads to seasonal epidemics and in some cases threatening pandemics casing significant morbidity and mortality. Respiratory viruses generally spread rapidly due to their mode of transmission which can be as simple as inhaling droplets containing the viral particles or through physical contact with contaminated items. The spread can be faster in densely populated areas or public gatherings. The transmission of virus can be very efficient and thus very difficult to control. The situation further deteriorates if the containment procedure is delayed or lacking. The world has already witnessed this in almost every influenza outbreak [1,2,3,4], as well as the outbreak of severe acute respiratory syndrome coronavirus (SARS-CoV) during the years 2002–2003 [5,6].

Vaccination is still the most efficacious method of preventing viral infections; however, this is still a challenge for many viruses, especially in highly mutating influenza viruses that require annual vaccine re-formulation. This situation often gets worsened since influenza viruses are known for cross-species spread from swine or birds [7,8] and infect humans when they have either low or no cross protective immunity [9]. Although there are studies that show advancements in the development of universal influenza vaccine (reviewed in [10]), yet the actual scenario is different than what was expected and none of these approaches thus far have shown clinical efficacy in large-scale human trials.

In case of viruses like respiratory syncytial virus (RSV), the vaccine development has major challenges such as the inadequate response to vaccination during immunization of very young infants and the circulation of the two antigenically distinct RSV groups (A and B) [11].

Current drug research and development has offered some specific biologically active molecules designed against targets that are responsible for disease progression. These molecules can efficiently block viral replication in infected individuals and therefore serves as promising therapeutic interventions against several respiratory viral infections. Of several such strategies, nucleic acid-based molecules have shown tremendous potential to block gene expression either during the transcriptional or posttranscriptional level [12]. These nucleic acid-based molecules have also been shown to have potential applications in cancer [13], neurological disorders [14], cardiovascular [15], inflammatory disorders [16], and infectious diseases [17,18,19,20]. One of the nucleic acid-based therapeutic agents, short interfering RNAs (siRNA), has been shown to have the potential to induce knock-down of mammalian genes at specific sequences without triggering the interferon response [21]. Similarly, other nucleic acid-based antiviral compounds such as ribozymes, deoxyribozymes (DNAzymes), antisense oligonucleotides, and aptamers have been developed that demonstrates potential applications in management of several respiratory virus infections in experimental conditions. Although all these strategies have problems of instability in vivo due to the nuclease activity and efficient delivery to the specific target cells, recent advancements have been made to overcome such problems by optimizations.

This review summarizes the present status of nucleic acid-based antiviral therapeutics and challenges in their delivery, especially in the context of respiratory viral infections.

## 2. Nucleic Acid-Based Therapeutics

Nucleic acid-based therapeutics is broadly comprised of the DNA-based therapeutics and the RNA-based therapeutics. These molecules offer sequence-specific cleavage of transcripts and have gained popularity in recent years due to their ability to downregulate disease-causing genes either through RNA interference or catalytic cleavage of the transcripts.

### 2.1. RNA Therapeutics against Respiratory Viruses

RNA molecules were long believed to only serve as messengers having information from genomic DNA. However, since the last two decades, small RNA molecules (∼20–30 nucleotides) have been evaluated as critical regulators of the expression and function of eukaryotic genomes. These small RNAs can be primarily categorized as short interfering RNAs (siRNAs) and microRNAs (miRNAs), both of which work by degrading the mRNA of specific genes with complementary nucleotide sequences, thus inhibiting their expression [22] and preventing translation.

#### 2.1.1. Short Interfering RNAs (siRNAs)

**Figure 1:**
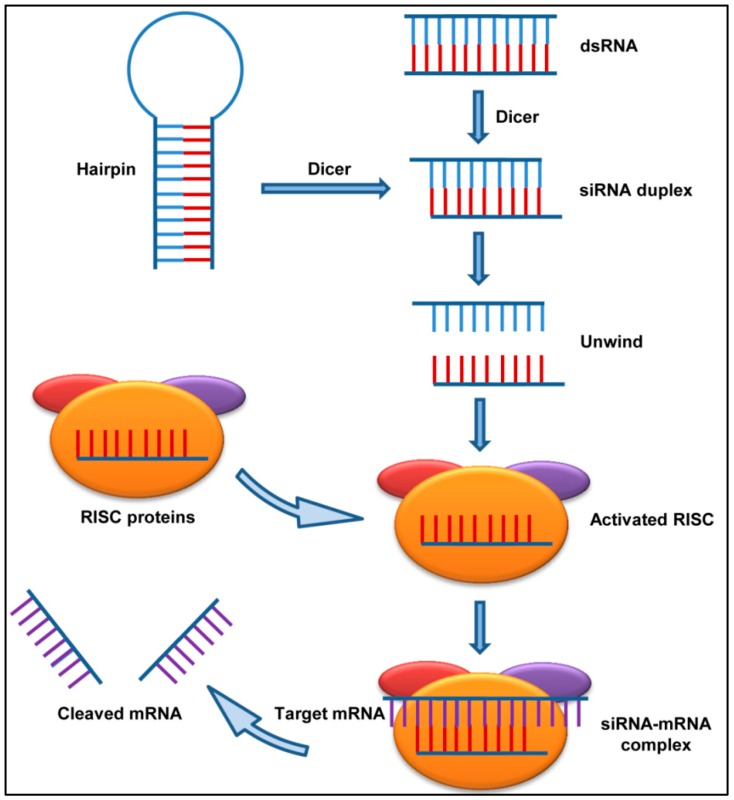
Mechanism of action of short interfering RNAs (siRNA): The presence of a double stranded RNA as a consequence of viral infection triggers RNA interference (RNAi). The host enzyme Dicer binds to double-stranded RNA (dsRNA) and cleaves it into short pieces of ∼20 nt called siRNA. One strand of the RNA associates with the RNA induced silencing complex (RISC) proteins and binds to the target mRNA. The mRNA is then cleaved by the nuclease activity of the RISC.

siRNAs and their role in the post-transcriptional gene silencing (PTGS) were discovered in plants by Balcombe’s group in 1999 [23] and in the roundworm Caenorhabditis elegans by Fire et al. in 1998 [24]. Years later, another group showed that the synthetic siRNAs could induce RNA interference (RNAi) in mammalian cells [21]. This discovery was one of the most significant advances in biology that led to potential usage of RNAi in biomedical research and drug development. The siRNAs are typically 21–23 nucleotide long double-stranded RNA segments that interfere with the expression of specific target genes with complementary nucleotide sequences by degrading their mRNA post transcription (Figure 1).

The long dsRNA introduced in cells directly or via any virus or transposons, is immediately recognized by RNA binding domain (RBD) of the enzyme Dicer, which contains an endonuclease catalytic domain. The dsRNA then gets cleaved into the short siRNA molecule which gets bound by a multiprotein component complex referred to as RISC (RNA induced silencing complex). One of the siRNA strands then guides and aligns the RISC complex on the target mRNA. Thereafter, the Dicer recruited Argonaute protein (which is also an RNase) slices the target mRNA thus causing gene suppression and consequent decrease in protein levels (Figure 1). The highly specific cleaving ability of the siRNAs makes them a potent prophylactic and therapeutic treatment option in targeting the disease-causing genes. However, optimization is required during siRNA design to avoid any off-target effects and minimize the possible side effects in the host cells.

#### 2.1.2. siRNAs against Respiratory Viruses

Bacteria and viruses cause respiratory infections in the airways [25]. Antimicrobial resistance has made treatment difficult for many infectious diseases including respiratory infections and RNAi therapeutics have proven to be effective against infections [26]. Since RNAi therapy target RNA, these are very advantageous over conventional small molecule drug used for therapy against respiratory diseases. Since the first time RNAi was used against P and F sequence of the respiratory syncytial virus (RSV) [27], there has been an increase in the number of studies related to the potential of RNAi technology in the treatment of other respiratory viral infections including influenza [28,29,30,31] and severe acute respiratory syndrome (SARS) [32]. RNAi molecules are designed specifically to target specific sequences of viral mRNA and thus can also be used efficiently against mutated viral strains when compared to other antiviral drugs.

Seasonal influenza viruses are one of the foremost public health concerns, especially influenza type A viruses that have been associated with the annual epidemics worldwide [33]. The recent 2009 H1N1 pandemic caused high morbidity [34,35] and mortality [36,37,38], and was a reminder that influenza viruses are highly unpredictable [3,39,40,41] and continuous efforts are needed to manage pandemic scenarios in short time. Antiviral drugs that are currently approved to treat influenza include the Adamantanes (M2 ion-channel protein inhibitors) and the neuraminidase inhibitors such as zanamivir and oseltamivir [42]. Few viral strains have now been found to be resistant to these drugs [43,44,45], thus emphasizing the need for urgent development of new antiviral drugs. Though the virus can sometimes inhibit the effect of host gene silencing, there has been studies using synthetic siRNA [46]. Due to the importance in viral replication and progression, the most commonly targeted viral regions are the nucleocapsid protein (NP), the polymerase basic protein (PB1), and the polymerase acidic protein (PA) which are highly conserved across different subtypes of influenza virus strains [47]. The first animal study using RNAi against influenza was described in 2003 where authors showed the targeted mRNA degradation using NP- and PA-specific siRNAs resulting in global inhibition of other viral RNA transcription. The reason for such significant inhibition is that the newly synthesized NP and PA proteins are indispensable for most influenza viral strains [48]. In another study, the polyethyleneimine (PEI) complex containing siRNA against NP and PA were administered intravenously to the mice either 3 hours (h) before or 5 h after influenza virus (Strain A/PR/8/34 (H1N1)) infection. The combination of these siRNAs enhanced the antiviral effect. The study also indicated that RNAi mediated by these siRNAs could partially control influenza virus even when administered after the viral infection in mice. The study also emphasized on long lasting effect of short hairpin RNAs (NP and PB shRNAs) expressed from DNA vectors [49]. These observations suggested that the siRNAs, when coupled with appropriate carriers and optimized formulations, may provide the basis for preventive treatment against influenza virus. Administration of siRNAs using lipid carrier to influenza-infected mice by hydrodynamic injection and intranasally has efficiently saved animals against highly pathogenic avian influenza A viruses (IAV) such as the H5N1, H7N7, and H9N2 subtypes [50]. Studies have also targeted the NS1 gene of influenza A virus and demonstrated a significant decrease in viral replication in experimental mice [51]. In a follow-up study from the same group, the *M1* gene of Influenza A virus was targeted using siRNA-Rz chimeric construct which lead to a considerable decrease in virus titer in experimental conditions [52]. There is a growing numbers of studies in search of potential usage of siRNAs both in vivo and in vitro models for the treatment of influenza [50,53]. Although there are few setbacks in the usage of these therapies due to the innate immune response rather than RNAi mediated viral inhibition, the advanced chemically modified RNAi molecules with a potential to trigger less immune response could be helpful in addressing this concern [54,55]. Some studies have also carried out siRNA mediated knockdown of cellular proteins. The authors targeted the Ran-binding protein 5 which resulted in the delayed accrual of the viral RNA polymerase complex in the infected cells thereby confirming that cellular genes along with viral genes, can also be targeted for therapeutic intervention in viral diseases [56].

RSV infection is one of the leading causes of respiratory distress in children [57,58] and in adults, the infection leads to self-limited upper respiratory illness [59]. However, premature infants and immunocompromised patients especially those with heart and lung disease can have severe RSV infection [60]. Current treatment plans for RSV are of limited benefit and include bronchodilators, corticosteroids, and antibiotics [57] for bacterial co-infections. To date, there is unavailability of a licensed vaccine available against RSV [61] thus presenting the urgent need to develop effective RSV therapeutics and vaccines to protect both the pediatric and the adult population. Since RSV replication takes place in respiratory epithelium, the nasal delivery of the RNAi molecule can be helpful in inhibiting RSV replication [62]. In a study in the year 2005, siRNA against viral phosphoprotein (P protein) was given intranasally to mice, with and without the help of transfection agent and was proved to be effective against both RSV and parainfluenza virus (PIV). The antiviral activity of the siRNA on the animal model was comparable to that in cell culture. The experiment also revealed competitive RNAi machinery when siRNAs against two different viruses were used in dual infection setting. It was observed that an excess of one siRNA toned down the inhibitory effect of the other. The study also suggested that if properly designed, low dosages of inhaled siRNA might prove to be an effective antiviral treatment against respiratory viral infections in humans. When compared to complexed siRNA, the naked siRNA showed approximately 70–80% efficiency. Moreover, the study also revealed that the administration of siRNA before RSV infection was more effective than treatment after infection [63]. Since siRNA to P protein could target only a few viral strains, another siRNA that could target viral nucleocapsid protein (N protein) was designed. The usage of this siRNA (ALN-RSV01) developed by Alnylam Pharmaceuticals not only decreased the viral load significantly in animal model but also the phase I clinical study of ALN-RSV01 proved to be successful with less side effects. The phase II trial has also been successful where ALN-RSV01 was given to the healthy adults in the form of nasal spray daily for two days prior to and for three days after RSV infection. The results showed that, compared to the placebo group, the subjects treated with ALN-RSV01 had a significantly lower number of RSV infections [64] showing the antiviral efficacy of ALN-RSV01. Reduced acquisition of the viral infection was observed within 3–4 days after virus inoculation and continued till the end of the study. The ALN-RSV01 also entered phase II b clinical trial in the RSV-infected lung transplant patients [65]. Though the marginal reported decrease in new or progressive bronchiolitis obliterans syndrome (BOS) in these patients was statistically non-significant, it was still promising and futuristic. ALN-RSV01 has not been associated with any significant adverse event in adult population; however, safety and efficacy of the drug is yet to be disclosed for the highly susceptible pediatric population. Since the usage of siRNAs targeted against cellular proteins required by influenza virus showed significant results, the same approach can be applied in case of RSV too. However, it should be kept in mind that these cellular genes/proteins should not be indispensable for cell survival. Among such proteins are the actin regulatory proteins, VASP (vasodilator-stimulated phosphoprotein) and Zyxin, and thus siRNA against these proteins can be used against RSV [66,67,68,69]. These proteins are non-essential for cell survival but they are required for maintaining normal physiological conditions; hence, only a short exposure of specific siRNAs can be afforded under critical conditions.

SARS-CoV is another major cause of respiratory distress. As there is no good vaccine or effective treatment for SARS-CoV infection, relying on RNAi therapy to counter the infection may prove to be an important tool to counter infection. Detailed description of the SARS-CoV genome allowed immediate advancement in the SARS-CoV RNAi based therapeutic. Clinical trials are underway, but some success has been achieved in cell culture models [70,71,72]. Identification of candidate nucleic acid paves the path for rational design of siRNAs against viral segments. Wang et al., in 2004, generated six antiviral RNAi activators that targeted various specific sites within the SARS-CoV genome [70]. pSR02 and pSR03, had similar antiviral effect against the ORF 1b sequence. In studies using Vero cell cultures and animal models, these RNAi activators decreased viral replication and weakened the cytopathic effects demonstrating high efficacy of the designed synthetic siRNAs against the virus [71]. The inhibitory effect of the siRNA designed against S sequence was remarkable [72]. Recently, the effect of these synthetic siRNAs with cognates in the ORF 1b and S regions of the SARS-CoV genome have also been investigated on murine and rhesus macaque models [32,73]. The otherwise unmodified short duplexes had dTdT overhangs at their 3′ ends. The intranasal administration of the dextrose soluble siRNAs to SARS-CoV-infected cells inhibited replication of SARS-CoV effectively [73]. In another study, siRNAs specific for protein responsible for viral entry (HCoV-NL63 S glycoprotein), showed a significant inhibition of viral replication in virus-infected cells [74].

Adenoviruses are also known to cause mild respiratory and gastrointestinal infections. Several studies have utilized siRNAs against adenovirus to inhibit their replication. In one such study, the authors designed several siRNAs against the early (*E1A*) and the late (hexon, *IVa2*) genes of adenovirus 5 [75] and showed variable degree of inhibition of virus replication. The study also revealed that silencing the late gene was more efficient than silencing the early gene in obstructing virus replication [75].

#### 2.1.3. Ribozymes (Rz)

**Figure 2:**
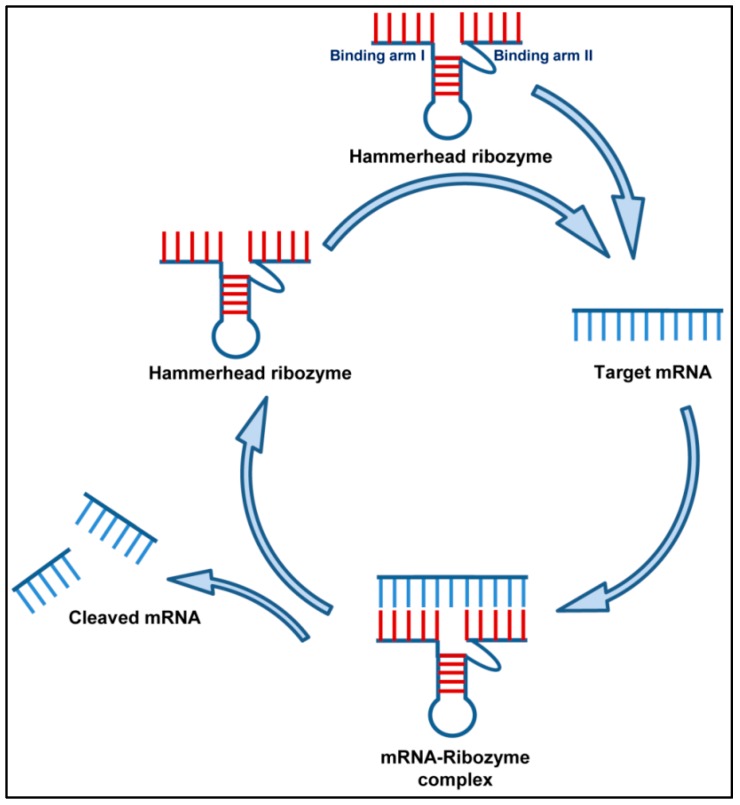
Mechanism of action of Ribozyme. A hammerhead ribozyme has two binding arms designed to bind to the complimentary RNA targets in a Watson–Crick pairing. The ribozyme binds to its target mRNA and makes an mRNA-ribozyme complex. The catalytic motif of the ribozyme then cleaves its target RNA into pieces, thus inhibiting gene expression. After cleaving the RNA target, the ribozyme becomes free again to enter into the next cycle.

Ribozymes are RNA molecules that have catalytic activity just like the DNAzymes. Since their discovery, the role of RNA catalysis has been shown in several biological processes such as the RNA splicing, RNA processing and the replication of RNA genomes [76]. The Rz occur in nature and mainly cleaves the phosphodiester bonds of nucleic acids. There are several classes of ribozymes, of which only the hammerhead and hairpin ribozymes have received a great deal of attention because of their smaller size. The hammerhead Rz has a 22-nt-long conserved catalytic core, that target RNA with NUX (N-any nucleotide and X-any nucleotide except guanosine) sequence, along with two flanked hybridizing arms (complimentary to target RNA) (Figure 2) [77]. Several studies have utilized the hammerhead Rz for catalytically cleaving the target RNA due to its high catalytic activity. The hammerhead and hairpin ribozymes in different studies have been used to significantly disrupt and reduce viral replication hence effectively inhibiting pre-genomic RNA levels of infecting viruses.

#### 2.1.4. Ribozymes against Respiratory Viruses

The propensity for high mutation in RNA viruses lead to the emergence of drug resistant strains which is a major challenge in antiviral chemotherapy. To overcome the toxicity and resistance concerns, naturally occurring molecules such as the ribozymes in the host cells are being extensively explored as potential therapeutic agents.

Tang et al., in their study, showed the ribozyme mediated inhibition of influenza virus infection in both in vitro and in vivo experiments [78]. They designed both the hammerhead and the hairpin ribozymes to cleave the viral RNA-segment 5 of influenza A virus and observed the effect of hammerhead Rz better than the hairpin Rz [78]. In another study by Motard et al., SOFA-HDV-Ribozymes were engineered as therapeutic agent to recognize conserved regions of the influenza virus sequences and to catalytically cleave the corresponding viral mRNA targets and disrupt viral replication [79]. Further characterization of the ribozyme’s antiviral effect in cell culture and in mice supported the prophylactic potential of SOFA-HDV-Ribozyme combinations as influenza anti-infective and served as novel strategy for antivirals against genetically and highly variable respiratory viruses [79]. A study also showed the utilization of external guide sequences (EGS) and ribonuclease P (a type of ribozyme) to inhibit the influenza virus production in mouse cells [80]. In yet another study, the Influenza virus PB1 gene was targeted by Rz directed to cleave the PB1 mRNA [81]. The target cells were made to express the designed Rz and its effect on inhibition of influenza A virus strains A/Singapore/1/57 and A/WSN/33 production and subsequent virus-specific protein synthesis was evaluated. The study showed a significant 93.5% inhibition in virus reproduction with designed Rz as compared to control cells [81]. In another novel mechanistic study, authors designed the siRNA-chimeric-ribozyme constructs to inhibit influenza virus replication [52]. Both the designed siRNA and hammerhead Rz were directed to cleave the M1 RNA of influenza A virus. The chimeric construct demonstrated a >80% protection against the virus challenge; however, the selectively disabled mutant constructs were far less effective [52]. In another follow-up study, the same group showed that effect of several Rz designed to target multiple sites in the M1 mRNA of influenza virus [77]. The study demonstrated that Dz132 and Rz163 when used together gave an enhanced cleavage effect than when they were used individual or additive effect [77]. The study also showed that antisense molecules designed to hybridize to specific sequences upstream and downstream of the Rz163 gave significant cleavage effect than Rz163 alone [77]. The ribozymes have also been reportedly used to generate recombinant influenza viruses [82]. The study dealt with transfection of plasmids carrying the human RNA polymerase I promoter and hepatitis delta virus ribozyme sequences in in vero cells in order to rescue influenza A viruses by reverse genetics [82].

A study performed on SARS-coronavirus using a chimeric DNA–RNA hammerhead ribozyme showed the usefulness of Rz in inhibiting virus replication upto 60% [83]. The authors further showed that the chimeric construct targeting SARS-CoV significantly reduced the expression of SARS-CoV RNA in 3T3 cells transfected with the recombinant plasmid, thus proving a feasible treatment option for SARS [83].

A virus infection in any target cells begins with the step of successful entry followed by trafficking in the cytosol before the viral genome reaches its destination. During these events, specific viral proteins interact with several host proteins in order to initiate signaling required for the infection. One such study focused on the identification of the most optimal Rz target sites within the fusion gene of Adenovirus E1A-Associated 300 kDa Protein [84] and suggested that Rz mechanism can also be exploited for studying the functionality of host proteins required for viral infections [84].

### 2.2. DNA Therapeutics against Respiratory Viruses

DNA was first isolated 150 years ago and identified by James Watson and Francis Crick 65 years ago. Initially, the only function attributed to DNA was to carry and pass genetic information from one generation to another. However, with advancements in science, DNA molecules got recognition for having a new role in the field of materials science [85].

#### 2.2.1. Deoxyribozymes (Dz)

**Figure 3:**
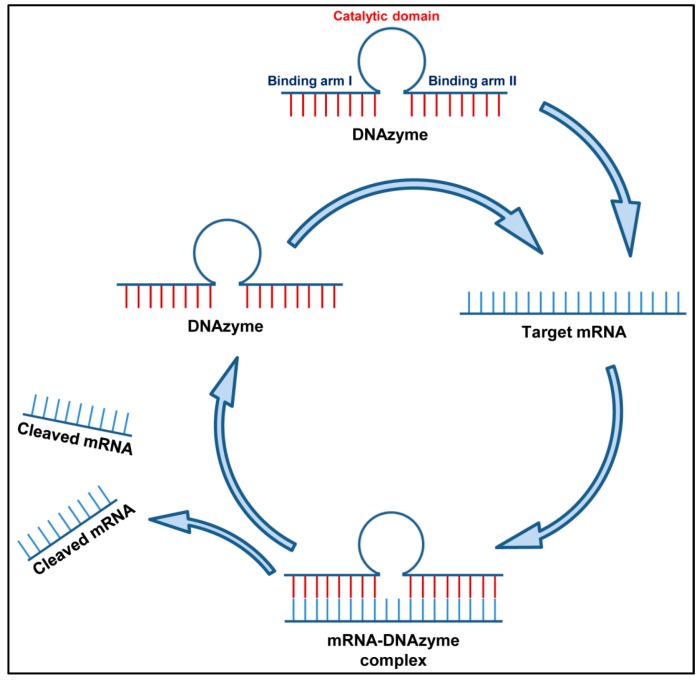
Mechanism of action of DNAzyme: A DNAzyme has two binding arms designed to bind to the complimentary RNA targets in a Watson–Crick pairing. The DNAzyme binds to its target mRNA and makes an mRNA–DNAzyme complex. The catalytic motif of the DNAzyme then cleaves its target RNA into pieces, thus inhibiting gene expression. After cleaving the RNA target, the DNAzyme becomes free again to enter into the next cycle.

Deoxyribozymes, also known as DNAzymes or DNA enzymes, are synthetic catalytic single-stranded deoxyribonucleic acid molecules that display precise substrate recognition and have the ability to cleave sequence-specific mRNA molecules with greater biological stability [86]. A Dz molecule has one central catalytic motif flanked by two arms. Both the arms (I and II) are designed complimentary to the target RNA molecule so that the designed Dz binds to it on a Watson–Crick basis (Figure 3). Of the several types of DNAzymes known to catalyze functions like RNA ligation [87], carbon-carbon bond formation [88] and the hydrolytic cleavage of DNA, the best characterized one is still the RNA-cleaving DNAzymes. DNAzymes have not been reported to occur naturally as DNA molecules are predominantly double stranded; however, Breaker and Joyce generated a DNAzyme de novo by an in vitro selection process in the year 1994. Further in vitro selection experiments generated two prototypes denoted as the “10–23” and the “8–17” RNA-cleaving DNA enzymes. The 10–23 Dz studied by several researchers, has been shown to cleave the target RNA between a purine and pyrimidine both under the in vivo and in vitro conditions. The 8–17 Dz also cleaves the RNA, however it is less popular because of its less established efficacy. The 8–17 Dz cleaves between A and G nucleotides and require a rG–dT wobble pair in the enzyme–substrate complex, located immediately after the cleavage site for its cleavage activity.

#### 2.2.2. DNAzymes against Respiratory Viruses

DNA-based drugs have notable advantages over currently available low molecular weight pharmaceuticals because of their specific recognition of molecular targets resulting in pinpoint action [89]. Several studies have demonstrated the effect of DNAzymes specifically targeting vital genes of influenza virus, RSV, and SARS-CoV to inhibit their replication in cell line and mice models.

Toyoda et al. investigated the use of 10–23 Dzs targeting PB2 mRNA to inhibit virus replication as potential therapeutics against respiratory infections caused by influenza A virus [90]. Dzs, especially those with longer substrate-binding domains, were able to inhibit influenza virus replication in cells. Modified anti-PB2 10–23 Dzs was similarly shown to inhibit influenza A virus. The most active analogues containing N3′–P5′ phosphoramidate linkages reduced the level of influenza A virus replication by 99% but demonstrated no activity towards influenza B virus [91]. Likewise, catalytically inactive mutants showed 20–50% inhibition perhaps due to their antisense action.

Several novel Dz and hammerhead ribozymes (Rz) have been constructed to cleave the conserved domains of the influenza virus M1 RNA as an antiviral strategy to reduce the infectiousness of the virus. A real-time polymerase chain reaction (PCR) study showed that both Dz and Rz can inhibit replication of the virus individually with a substantial decrease (54%) in virus replication when used simultaneously. The authors concluded that combining catalytic Dz and Rz with antisense molecules lead to a more efficient gene suppression and complete inhibition of virus replication in host cells [77]. Various Dz have also been studied for activity against influenza B virus targeting the BM2 ion channel protein. BM2 has a highly conserved sequence and is crucial during the uncoating process of the influenza virus. One of the Dz (Dz209) demonstrated significant intracellular reduction of BM2 gene expression in transient-expression system and provided protection against influenza B virus challenge in Madin–Darby canine kidney cells [92]. In a follow-up study from same group, the Dz114, among several others, exhibited 70% inhibition of influenza A virus M2 gene expression as shown by the PCR and Western blot analysis. The activity of the designed Dz was further shown to be dependent on the Mg (2+) in a dose-dependent (10–50 mM) manner [93].

RSV has also been targeted using DNAzymes. A series of Dzs were designed and synthesized to silence RSV N, M2 and F genes. One Dz (DZn1133) cleaved the RSV RNA in vitro, inhibited the transcription and expression of F viral gene, reduced the RSV production by about 7 logs and protected more than 90% of RSV-infected Hep-2 cells from the cytopathic effect when used at the concentration of 8 µM. Additionally, 10 wild RSV strains (including both subgroups A and B) isolated from patients showed significant suppression by DZn1133 with higher anti-RSV activity than that for ribavirin (1-β-d-ribofuranosyl-1*H*-1,2,4-triazole-3-carboxamide) or an antisense oligonucleotide (ODN) complementary to the same region of the N RNA [94]. Treatment with Dzn1133 reduced viral plaque formation in the lungs of RSV-infected BALB/c mice. Additionally, it was observed that the designed Dz significantly reduced the viral mRNA expression, alleviated the airway inflammation, and reduced the leukocyte counts in the bronchoalveolar lavage fluid of RSV-infected mice. The DZ1133 showed a dose-dependent (0.2–0.8mg) antiviral effect and was observed to be more efficient than the antisense oligonucleotide mediated inhibition of gene expression even though levels of cytokines such as the TNF-alpha, IFN-gamma, IL-12, and IL-10 induced by the RSV infection were unaffected. The overall results demonstrated that DZ1133 can be used as a potential therapeutic agent against infections with both subgroups A and B of RSV in vivo [95].

SARS-CoV has also been targeted using Dz molecules. The use of a Dz to target the 5′-untranslated region (5′-UTR) of a highly conserved fragment in the SARS-CoV genome to suppress viral replication has been reported. A study showed the efficacy of a mono-DNA enzyme (Dz-104) possessing the 10-23 catalytic motif, both in vitro and in cell culture experiments. The Dz-104 showed an in vitro cleavage of the SARS-CoV RNA substrate and reduced the expression of the SARS-CoV 5′UTR-eGFP fusion RNA in the mammalian cells. The results provide a basis for the potential therapeutic usage of DNA enzymes to combat SARS infection [96].

#### 2.2.3. Antisense Oligonucleotides (ASOs) 

Antisense oligonucleotides are small synthetic pieces of single-stranded DNA that are normally 15–30 nucleotides in length. ASOs specifically bind to complementary DNA/RNA sequences by Watson–Crick hybridization and once bound to the target RNA, inhibit the translational processes either by inducing cleavage mechanisms or by inhibiting mRNA maturation [97]. The use of ASOs was first reported by Zamecnik and Stephenson in 1978 as a potential antiviral therapeutics. They utilized a phosphodiester oligodeoxynucleotide composed of 13 nucleotides (a 13-mer) that was designed to block Rous sarcoma virus replication [98,99]. Since then, ASOs ability to selectively inhibit gene expression has generated noteworthy enthusiasm in the scientific and medical community because of its specificity and the breadth of its potential applications as therapeutic agents. An extensive range of oligonucleotide analogs has become available over the past decade and this lead to target validation and development of ASO-based antiviral agents whose efficacy have been reported against various virus types, both in vitro as well as in vivo [100]. For ASOs to be used empirically, modifications of DNAs or RNAs were needed to retain hybridization capacity at the same time increasing stability. Major alterations have been introduced in the phosphodiester bond, the sugar ring, and the backbone to result in three generations of nucleic acid analogs for the synthesis of ASO oligomers. ASO-based antiviral agents are specifically designed to block the translational processes either by (i) ribonuclease H (RNAse H) or RNase P mediated cleavage of mRNA or (ii) by sterically (non-bonding) blocking enzymes that are involved in the target gene translation [97].

#### 2.2.4. Antisense Oligonucleotides against Respiratory Viruses

Antisense oligonucleotides have been studied extensively against several respiratory viruses with promising results.

The earliest studies using oligonucleotide (‘oligo’) to inhibit synthesis of virus-specific proteins, including influenza, in MDCK cells were reported in the 1990s. Researchers observed that the modified oligos could effectively suppress the influenza A/PR8/34 (H1N1) virus production [101]. Since then several other ASOs have been synthesized and studied for efficacy against influenza. Ge et al. showed that siRNAs specifically designed to target the conserved regions of the viral genome can potently inhibit influenza virus production in cell lines (Vero, MDCK) as well as embryonated chicken eggs [48]. Wu et al. showed that in vivo treatment with three doses of RNA oligonucleotides conferred significant protection to the infected chickens from H5N1 virus-induced mortality at a rate of up to 87.5%. The authors used mixed RNA oligonucleotides targeting the *NS1* gene to show a significant reduction in the plaque-forming unit (PFU) and viral RNA levels in the lung tissues of the infected animals by plaque assay and real-time PCR analysis. Their study demonstrated that RNA oligonucleotides targeting at the viral NS1 gene could potently inhibit highly pathogenic avian H5N1 influenza virus reproduction and thus, could potentially be used as prophylaxis and therapy for H5N1 influenza virus infection in humans [102]. In another study, Gabriel et al. used three peptide-conjugated phosphorodiamidate morpholino oligomers (PPMOs), to selectively target the translation start site region of PB1 or NP mRNA or the 3′-terminal region of NP viral RNA, to prevent virus replication in MDCK cells. The study further utilized the primer extension assays to show that treatment with any of the effective PPMO markedly reduced the levels of mRNA, cRNA, and vRNA [103]. Another study by Duan et al. used a novel antisense oligonucleotide (IV-AS) specifically designed against the 5′-terminal conserved sequence found in all the eight viral RNA segments of influenza A virus. They monitored the activity of IV-AS both in vitro in the MDCK cells and in vivo using a mouse model. IV-AS was administered intranasally to the H5N1-infected mice once daily for 6 days starting 6 h post-infection. IV-AS, at 50% effective concentration (EC50) ranging from 2.2 to 4.4 µM, inhibited influenza A virus induced cytopathic effects in MDCK cells. IV-AS was also effective against H5N1 virus in preventing death, reducing weight loss, reducing lung consolidation and decreasing the lung virus titers [104]. Lupfer et al. showed that antisense-PPMOs, delivered through the intranasal route, were able to inhibit the replication of equine influenza A virus A/Eq/Miami/1/63 (H3N8) in mice by over 95% compared to the controls [105]. In another study, a group of authors designed antisense oligonucleotides against the common 3′ NCR of segments of the IAV genome to inhibit its replication [106]. The AS molecules demonstrated a drastic reduction in the cytopathic effect caused by A/PR/8/34 (H1N1), A/Udorn/307/72 (H3N2), and A/New Caledonia/20/99 (H1N1) strains of IAV for almost 48 h post-infection. The same AS molecule protected mice against all the strains of influenza virus [106]. Giannecchini et al. tested phosphorothioate oligonucleotides (S-ONs) obtained from the packaging signals in the 3′ and 5′ ends of the PB2 vRNA against influenza virus in vitro. The 15-mer S-ON (designated 5–15b) derived from the 5′ end of the PB2 vRNA, and complementary to the 3′ end of its coding region (nucleotides 2279–2293), proved noticeably inhibitory. Similar to other related studies, the antiviral activity of 5–15b was also observed to be dose- and time-dependent; however, it was independent of the cell substrate and multiplicity of infection used in the study [107]. In another follow-up study, Giannecchini et al. investigated whether analogous inhibitory S-ONs targeting PB1 and PA gene segments could be identified and if virus can develop resistance to S-ONs. The authors observed that the 20-mer S-ONs reproducing the 5′ ends of *PB1* and *PA* gene segments exerted a dominant antiviral activity against several influenza A virus subtypes in MDCK cells. Their findings suggest that the packaging signal at the 5′ end of the PB2 vRNA can be a potential therapeutic target for the design of novel anti-influenza compounds. Also, antivirals against this region could be beneficial owing to less changes of mutation in these viral genes [108].

Recently, Lenartowicz et al. designed and tested 2′-*O*-methyl and locked nucleic acid antisense oligonucleotides (ASOs) to specifically target the internal regions of influenza A/California/04/2009 (H1N1) viral RNA segment 8. Of the 14 designed and tested ASOs, 10 showed significant inhibition of viral replication in MDCK cells. The ASOs were 11–15 nucleotides long and demonstrated varying inhibition ranging from 5- to 25-fold. The designed ASOs were very specific for IAV and showed no inhibition of influenza B/Brisbane/60/2008 (Victoria lineage). The combinations of ASOs slightly improved anti-influenza activity. These studies show that ASOs can be designed to be accessible to IAV RNA in regions other than the panhandle formed between the 5′ and 3′ ends [109].

RSV has also been targeted by several designed ASO molecules. Jairath et al., in their study investigated the use of oligodeoxyribonucleotides to inhibit RSV replication in cell culture. Human epithelial type 2 (HEp-2) cells were infected with RSV strain A2 and treated with the designed oligonucleotides. A 0.5–1 µM 50% effective concentration (EC50) values were obtained for the designed antisense oligonucleotide targeted to the start of the viral NS2 gene. The ELISA and PT-PCR analyses showed that all the oligonucleotides inhibited virus antigen production and demonstrated sequence specific depletion of the genomic RNA target. The target RNA was observed to be cleaved at the specific antisense oligonucleotide binding site. The results suggest that antisense oligonucleotides could have therapeutic value against RSV infections [110]. PPMOs have the ability to readily enter cells and interfere with viral protein expression through steric hindrance of the complementary RNA. Lai et al. designed two antisense PPMOs to specifically target the 5′-terminal region and translation start-site region of RSV L mRNA [111]. Both PPMOs demonstrated minimal cytotoxicity when tested for anti-RSV activity in two human-airway cell lines. One PPMO (AUG-2), reduced the viral titers by >2.0 log10. Intranasal administration of AUG-2 in BALB/c mice before the RSV infection showed a reduction in viral titer of 1.2 log10 in lung tissue at day 5 post-infection, and further reduced pulmonary inflammation at day 7 post-infection. The overall results show that PPMO has a potent anti-RSV activity and the potential to be a therapeutic regimen against RSV infections [111].

Neuman et al. designed specific PPMOs against specific sequences in the SARS-CoV (Tor2 strain) genome [112]. The PPMOs were analyzed for their capability to inhibit infectious virus production and were further investigated to determine the function of conserved vRNA motifs and their secondary structures. Several virus specific PPMOs along with a random-sequence control PPMO were designed that showed low inhibitory activity against SARS-CoA. The virus-targeted PPMOs further reduced the cytopathology due to viral infection and reduced cell-to-cell spread because of reduction in viral replication. The active PPMO were found to be most effective when administered any time prior to the peak viral synthesis and exerted a sustained antiviral effect in the culture medium. The study demonstrated the antiviral effects in vitro for the PPMO designed complementary to the AUG translation start site region of a murine coronavirus replicase [112] and suggested a therapeutic potential of ASOs against coronavirus infection [113]. In another study, Ahn et al. evaluated the antiviral effects of antisense peptide nucleic acids (PNAs) targeting a highly conserved RNA sequence on the programmed −1 ribosomal frameshifting (−1 PRF) that is utilized by eukaryotic RNA viruses [114]. Cells transfected with a SARS-CoV-replicon, treated with the PNA (50% inhibitory concentration of 4.4 µM) fused to a cell-penetrating peptide (CPP), showed a significant suppression of the replication of the SARS-CoV [114].

### 2.3. Aptamers

**Figure 4:**
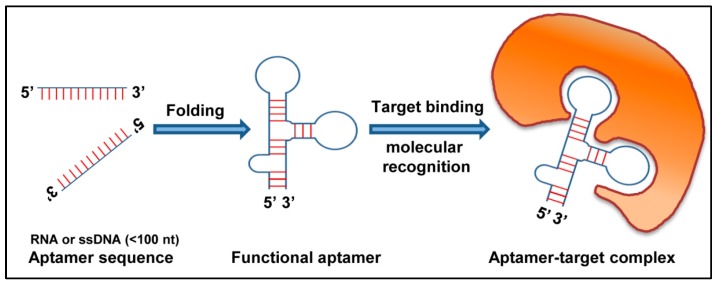
Schematic representation of action of aptamer: An aptamer folds into a three-dimensional structure. The functional aptamer then recognizes and binds to its target molecule resulting in a stable aptamer-target complex.

Aptamers are 20–90 nucleotides long, synthetic single strand nucleic acid molecules (DNA or RNA). They are designed to bind to various organic or nonorganic molecules—ranging from single atoms to a wide range of proteins (Figure 4). Aptamers are highly specific to target molecule and are generated by the SELEX (systematic evolution of ligands by exponential enrichment) method [115]. For the last 20 years, aptamers have been used as a diagnostic tool for the treatment of viral diseases.

#### Aptamers against Respiratory Viruses

Owing to pandemic nature of the disease and constant mutation in virus, identification of different subtypes becomes essential. In most cases antibodies are used to distinguish influenza A and B but most successful probes to distinguish different subtypes are aptamers. That is why aptamers to most common subunits of the virus are being used to develop sensors for influenza detection. Hemagglutinin (HA), is influenza viral surface glycoprotein responsible for fusion of virus with the host cell. In case of influenza, at least 18 different HA antigens has been identified which are not only responsible for diagnosis but identification of different influenza types and subtypes. Since 2004, more than 40 DNA and RNA aptamers against recombinant hemagglutinins (H1, H3, H5, H9, and HA from influenza virus B) and to whole viruses (H5N1) [116] have been constructed. Misono and Kumar used SPR-based SELEX to design and select an RNA aptamer and showed its activity against HA of A/Panama/2007/1999 (H3N2) [117]. Gopinath et al. constructed two targeted RNA aptamers, P30-10-16 and A-20, used to differentiate influenza type A from type B and closely related strains of the influenza subtypes [118,119]. RNA aptamer P30-10-16, was found to be 15 times more specific to H3N2 of influenza A virus than to any conventional anti-HA monoclonal antibody [119]. Subsequently, several aptamers were constructed to detect influenza virus type A (H1N1 and H3N2), as well as avian influenza virus, H5N1 [120,121]. These aptamers can prove to be helpful in diagnosing and distinguishing all the dangerous influenza strains with potentially high epidemiological risk. In 2013, Wang and Li generated biosensor based on quartz crystal microbalance (QCM) technique where they modified DNA aptamer by cross linking it to quartz particles to detect avian influenza virus more specifically [120]. Similarly, Bai et al. 2012 also suggested usage of an SPR aptasensor directed against avian influenza H5N1 [122]. Later on, the impedance-based aptasensor with microfluidics chips, with a lower detection limit than the SPR-based aptasensor and the same sensitivity as the QCM aptasensor, were used to detect H5N1 avian influenza virus [123]. Recently, Nguyen et al. used a SPR based sandwich type sensor to detect H5Nx viruses [124]. The sensitivity of this dual aptamer-based sensor increased many folds when the secondary aptamer was conjugated with gold nanoparticles. These chemical modifications of aptamers reduce selection time and increase their biostability in vivo. The early diagnosis and identification of subtypes, help target viral strains specifically and rationally.

Aptamers have been developed against influenza for therapeutics purposes. Many of these aptamers developed against HA could prevent the entry of the virus to the cells by blocking hemagglutinin activity which could be measured in vitro using hemagglutination inhibition assay. The usage of DNA aptamer A22 showed reduction of 95% of viral infection when administered concomitantly with the viral infection in the mice model. A22 was less effective when it was given prior to the infection [125]. In 2014, Musafia et al. used the modified A22 which had 10–15 times more potent antiviral activity in animal models than A22 aptamer. Many aptamers targeting other subunits important for viral replication and progression have also been studied [126]. Woo et al. successfully studied a DNA aptamer against NS1 subunit. This particular aptamer maintained the cell viability and induced IFN-β production while inhibiting the viral replication [127]. Later on, aptamers against subunits of polymerase complex of influenza virus, PA, PB1, and PB2 were also studied; and finally, the aptamers with a 50% inhibitory concentration (IC50) around 10 nM was identified that exhibited cross-protection against infections of H1N1, H5N1, H7N7, and H7N9 influenza viruses [128].

Few aptamers targeting host cell factors important for viral replication have also been studied. ApPABP7 and ApPABP11 aptamers which inhibit viral protein–host translation factor interactions have been useful against influenza [129]. Similarly use of a specific RIG-I aptamer efficiently blocks the replication of influenza and other viruses in infected cells through IFNα/β immune responses [130,131].

In case of yet another respiratory virus SARS, nucleocapsid (N) protein, one of the most abundant proteins, is the target choice for developing aptamers. Ahn et al. have developed a nanoarray aptamer chip with the RNA aptamer which can proficiently detect SARS nucleocapsid protein at a concentration as low as 2 pg/mL [132]. Likewise, ssDNA aptamer proficiently detects N protein by Western blot [133]. RNA aptamer ES15 used to target nsP10 (nonstructural protein 10) by Jang et al. inhibited viral enzyme activity in a dose-dependent manner (with IC50 = 1.2 nM) [134]. Yet another study showed that DNA aptamers against the non-structural nsp13 protein, inhibiting helicase activity of nsp13 protein required for SARS viral replication [135].

A recent study on RSV demonstrated that aptamers can be selected based on the rationally designed SELEX protocol against whole virus and that the specific aptamers can potentially be utilized for detecting the respiratory syncytial virus [136].

## 3. Methods of Delivery and Challenges

RNA interference mediated by tools like shRNA, ribozymes, and DNAzymes has emerged as an important technology not only to study gene functions but also for therapeutic purpose to manage viral infections and restrict the growth of cancerous cells. The biggest hurdle in the use of these tools is the in vivo delivery to target cells. Major challenges in in vivo delivery of the nucleic acids include their immunogenicity, enzymatic digestion in body fluids, phagocytosis by the immune cells, and renal clearance. Moreover, highly charged nature of nucleic acids does not allow them to cross the cell membrane by free diffusion. Intracellular challenges to the delivery of these molecules include endosomal and lysosomal escape in the cell cytoplasm. Several strategies have been developed for efficient in vivo delivery of nucleic acids which include use of both viral as well as non-viral vectors as the delivery vehicles.

With reference to respiratory viruses, numerous studies have been reported for non-viral vector mediated in vivo delivery of siRNA and ribozymes targeting the respiratory virus genome. Cationic systems like liposomes and polymers have been successfully used for systemic administration of siRNA molecules. Liposomal vectors being positively charged can efficiently form a complex with negatively charged effector nucleic acids on one side and interact with negatively charged cell membrane on the other side. The size of the vector also needs to be kept below 100 nm to prevent renal clearance after systemic administration. A polymer, polyethyleneimine (PEI), could be used as a carrier of shRNA targeted to mRNA encoding PA, PB1, and PB2 protein of influenza A virus. It was observed that shRNAs cloned in pSilencer2.1-U6 puro plasmid vector had a prophylactic effect on the mice infected with A/WSN/33 (H1N1) when delivered as shRNA-jetPEI complex through intra-tracheal route. However, these complexes could not show any therapeutic efficacy [137]. Another study was done on siRNA designed against gene encoding phosphoprotein of RSV. It was shown that siRNA delivered through intranasal route prior to infection, either naked or in complex with transfection reagent viz. TransIT-TKO reagent, could significantly reduce the viral titer and disease outcome in mice. However, again the effect of siRNA was very low when delivered after virus infection [63]. Alnylam Pharmaceuticals translated the results of study done by Bitko et al. into human trials to evaluate the antiviral activity of the siRNA under the name ALN-RSV01 which has crossed the Phase 2a clinical trial and found to be safe when delivered to healthy human individuals as nasal spray (aerosols) [65]. There are several other reports showing the efficacy of intranasal administration of siRNA in complex with transfection reagents to reduce viral titer in lungs of infected mice and to relieve the symptoms [50,138]. However, administration of siRNA complexed with polyethyleneimine or other transfection reagent like lipofectamine through intravenous route has also been shown to exert inhibitory effect on the virus replication in lungs of treated mice leading to more than 30% inhibition in the lung virus titer [49,53,139,140]. In the study done by Tompkins et al., the direct intravenous injection of siRNA-oligofectamine in PBS aided the effect of intranasal inoculation of the siRNA complex (targeted to nucleoprotein and acidic polymerase gene) thus reducing the pathogenicity of highly virulent influenza A virus strains [50]. Though PEI and other transfection reagents have been shown as effective carrier of RNAi tools in mice model, these are not feasible for clinical use because of their proinflammatory effect. Li et al. demonstrated that siRNA complexed with carriers like D5W solution and Infasurf solution is safe for clinical use. Intra-tracheal administration of siRNA-D5W solution complex targeted to spike coding gene and NSP12 region of SARS coronavirus (SCV) genome showed efficient inhibition of SCV replication and suppression of SARS like symptoms in monkey model [32].

Apart from the above-mentioned polymer or lipid-based delivery of shRNA, therapeutic RNA molecules can also be delivered through intranasal route in complex with chitosan nanoparticles. In a study done with chitosan nanoparticles, it was shown that intranasal inoculation of chitosan complexed with plasmid encoding shRNA targeted to NS1 gene of RSV could reduce RSV infection and lung pathology in rat model. The complex was also successful in modulating the immune response to RSV infection [141].

The role of ribozymes as therapeutic tool was shown by Motard et al. who designed SOFA-HDV-Rzs targeted to all the mRNA except that of hemagglutinin and neuraminidase gene of influenza A virus. The ribozymes targeted to conserved regions of mRNA corresponding to nucleoprotein and non-structural gene of influenza A virus were found to inhibit virus replication in the lungs of treated mice when delivered intra-nasally in complex with in vivo-jetPEITM. Although the extent of reduction in viral titer in the lungs of infected mice was low, the ribozymes could significantly affect the clinical outcomes of the infected mice [79]. In the same year, another study revealed an approach to manage respiratory syncytial virus (RSV) infection using ribozymes. The ribozyme was designed against the *L* gene mRNA of RSV and expressed via an inducible RSV minigenome replicon system which generated pseudovirus particles (defective RSV particles) expressing therapeutic ribozymes. The minigenome system could be induced by actual RSV infection and had the potential to inhibit virus replication when delivered intra-nasally via pseudovirus particles as delivery vehicles [142].

Viral vectors for delivery of RNAi tools for respiratory viruses are relatively less discussed. Baculovirus is one such vector. It is an insect virus vector known for its safety and high packaging capacity. The vector is considered safe as the recombinant baculovirus is replication incompetent in mammalian cell and does not produce immunogenic viral proteins. It is reported that recombinant baculovirus vector could exhibit transient but effective expression of shRNA targeted to influenza A and B virus under in vitro condition. The duration of expression could be increased by insertion of Epstein–Barr virus sequences into the vector [143]. The use of this vector for in vivo expression of RNAi tools needs to be examined.

Although RNAi has received significant attention in the basic and applied research related to biological systems, the RNAi associated immune stimulation may pose hurdle in clinical applications. The possibility of innate immune response induced by siRNAs/shRNAs or its delivery vehicles is yet another obstacle that needs to be addressed very carefully [144,145,146]. Recent studies have shown that advanced chemically modified RNAi molecules can be designed to trigger less immune response. Furthermore, the potential benefits of immunostimulatory siRNAs can be beneficial in case of viral infections and antagonistic cancers (reviewed in [147]). In spite of associated innate immune responses, the nucleic acid-based strategies still hold a lot of importance and most of them are currently undergoing various phase of clinical trial. They are not only shown to be effective against viruses, but they have also been used to target non-viral diseases (Table 1).

## 4. Conclusions

Respiratory viruses have been a global concern for decades and are the leading cause of morbidity and mortality in both children and adults. Some of these viruses show seasonality in pattern of outbreaks [150] and can be managed if proper precautions and preventive measures are initiated in time. The major ones, influenza and RSV, are the leading causes of respiratory disease and a continuous surveillance system is required to understand their changing pattern year after year. They both have coinciding seasonality which aggravates the scenario. Although several studies have focused on techniques that can help diagnose these viral infections in a short time [35,151,152,153], and symptomatic treatments are available, prevention is still better than cure. Vaccination is considered the most effective tool; however, the need for annual vaccine formulation against highly mutating influenza viruses is a reason to continuously try to develop novel strategies [154] to combat frequent epidemics. RSV does not have a successful vaccine yet [61], and only a few vaccine candidates are in clinical trials [155].

Nucleic acid-based therapeutics gained popularity because of its ability to target genes and inhibit translation. Strategies like antisense technology, ribozymes, DNAzymes as well as the RNAi-based therapeutics have greatly improved in the recent years. New studies have also developed improved strategies for overcoming the issues relating to instability by utilizing various types of modifications such as phosphorothioate (PS) DNA or 2-O-methyl-RNA (OMe) which proved to be more stable than normal DNA/RNA oligonucleotides. Several pathogens, including viruses, have been targeted utilizing one of these nucleic acid based silencing strategies. Numerous studies conducted in experimental animal models as well as in cell culture systems have evaluated the efficiency and efficacy of these molecules in post-transcriptional silencing of crucial viral genes to obstruct protein translation and inhibit viral replication. Some of these strategies have already made their place in clinical trials.

The efficacy of the nucleic acid-based molecules is dependent on their expression, stability, and accessibility to the target sites. If properly designed and modified, all these nucleic acids may have significant inhibitory effect on relevant target but combination of more than one type of therapeutic can always show synergistic effect against the virus propagation in host cells.

Although the discussed therapeutic nucleic acids are highly specific for their target and are optimized for nil off-target effect, hence, their side effects including effect on cellular metabolism is expected to be negligible. The risk factor associated with these molecules can be their delivery vehicles especially the viral vectors but if appropriately chosen, the related risk can be minimized.

The available literatures on such strategies show tremendous potential; however, the development of such molecules is not very simple and requires extensive optimizations in respect of their stability and modes of delivery. Any new discovery of antiviral compounds brings with it associated challenges; still, the effort of such discoveries is essential and potentially offers more effective and better tools to manage viral infections.

## Figures and Tables

**Table 1:** List of nucleic acid therapies in clinical trials [148,149].

Nucleic Acid Therapies	Disease	Company
**Antisense**		
Vitravene	Retinitis	CIBAVision, ISIS Pharmaceuticals
Kynamro	Hypercholesterolemia	Genzyme
Anti-c-MYC	Cardiovascular restenosis	Phase II AVI Biopharma
EPI 2010 (AS against adenosine A1 receptor)	Asthma	Phase II EpiGenesis Pharmaceuticals
Genasense (AS against BCL2)	Hematological malignancies Solid tumors, Phase III	Genta
GTI 2040 (AS against ribonucleotide reductase)	Solid tumors, Phase I and II	Lorus Therapeutics
HGTV (AS against HIV)	HIV, Phase II	Enzo Biochem
CpG molecules	Solid tumors Infectious diseases, Phase I/II	Coley Pharmaceutical Group
**Aptamer**		
Macugen™ (pegaptanib sodium), an anti-VEGF RNA aptamer	Diabetic Macular Edema (DME), Phase III	Eyetech Pharma
ARC1905 (Anti-C5 Aptamer), Zimura®	Dry ARMD, Phase II	Ophthotech Corp
E10030 (Anti-PDGF Pegylated Aptamer, Fovista®)	ARMD, Phase III	Ophthotech Corporation
ISIS 3521 (PKC-α) (AS) ISIS 5132 (c-RAF) ISIS 2503 (h-RAS) G 3139 (BCL2) GEM 231 (PKA)	NSCLC, NHL, Phase III Solid tumors, Phase II NSCLC, Phase II NHL, Phase II/III PKA, Phase II	ISIS Pharmaceuticals
**Ribozyme**		
Angiozyme (Ribozyme against VEGFR1)	Breast and colon cancer, Phase II	Ribozyme Pharmaceuticals
Heptazyme (Ribozyme against HCV) Herzyme (Ribozyme against HER2)	HCV, Phase II Breast and ovarian cancer, Phase I	Ribozyme Pharmaceuticals

AS: antisense; ARMD: age-related macular degeneration; HCV: Hepatitis C virus; BCL2: B-cell lymphoma protein 2; CpG: unmethylated CpG dinucleotides; NHL: non-Hodgkin’s lymphoma; NSCLC: non-small-cell lung cancer; VEGFR1: vascular-endothelial-growth-factor receptor 1.
